# Editorial: Stress-induced psychopathology: from mechanisms to interventions

**DOI:** 10.3389/fpsyg.2026.1889052

**Published:** 2026-07-07

**Authors:** Anson Kai Chun Chau, Guangzhe Frank Yuan, Hong Wang Fung

**Affiliations:** 1 Department of Psychiatry, School of Clinical Medicine, Li Ka Shing (LKS) Faculty of Medicine, The University of Hong Kong, Hong Kong, Hong Kong SAR, China; 2 School of Education Science, Leshan Normal University, Leshan, China; 3 School of Nursing, The Hong Kong Polytechnic University, Hong Kong, Hong Kong SAR, China; 4 Mental Health Research Centre, The Hong Kong Polytechnic University, Hong Kong, Hong Kong SAR, China

**Keywords:** resilience, risk factors, stress, stressors, trauma

The diathesis-stress model provides a theoretical framework for understanding how stress interacts with pre-existing vulnerabilities to shape the development of psychiatric disorders. This model emphasizes that, while genetic, neurobiological, and temperamental predispositions explain individuals' susceptibility, acute or chronic stressors serve as triggers for symptom onset and exacerbation across a range of psychopathologies, including depression, anxiety, schizophrenia, and trauma-related conditions such as dissociative disorders and post-traumatic stress disorder (PTSD). Stressful events and exposures range from DSM/ICD-defined PTSD trauma (e.g., Criterion A events such as assault or disasters) to subtler forms of adversities such as social exclusion, discrimination and neglect. However, the psychopathological pathways from stress to the development of various mental health symptoms and disorders are not yet fully understood. A more comprehensive understanding of these pathways would facilitate the development of targeted interventions for the improvement of mental health in both clinical and non-clinical populations.

This Research Topic, consisting of 16 articles, aims to reveal that stress-induced psychopathology emerges as a multilevel process in which genetics, neurobiological systems, developmental vulnerability across life course, affective and (social-)cognitive deficits, environmental adversity, and treatment contexts interact to determine the onset, maintenance, and recovery of mental health symptoms. Using a range of methodologies, these articles illuminate putative mechanisms linking stress to a range of mental health difficulties. The articles are organized into the following themes:

## Theoretical models of stress-related psychopathology

Two articles in common suggest stress as a biopsychosocial process operating across neurobiological, autonomic, cognitive-affective, and interpersonal levels. First of all, Duller et al. [*Everyday stress-induced neurobehavioral dysregulation: therapeutic targets for management with Neurexan*] conceptualized daily-life stress should be understood through the Research Domain Criteria (RDoC) framework as affecting negative valence and arousal/regulatory systems. The article emphasizes that ordinary, repetitive stress can lead to clinically significant dysregulation, such as changes in cortisol, autonomic nervous system responses, sleep, and functional connectivity patterns, that may amplify emotional and behavioral dysregulation. The key implication of this review is that intervention strategies (such as Neurexan) should target the RDoC domains most disrupted by daily-life stress.

Aligning with the integrated biopsychosocial model of stress-related psychopathology, Garcia-Toro and Gomez-Juanes [*Schismogenesis in anxiety spectrum disorders: a biopsychosocial perspective*] argued that anxiety-spectrum disorders can be understood as a biopsychosocial dissociation driven by “schismogenesis,” the runaway feedback across neurobiological, psychological, and social systems that disrupt integration and creates rigid hyperactivity and hypoactivity across systems. They reframe anxiety disorders through complex systems (or cybernetics), suggesting psychopathology may emerge when reciprocal feedback overwhelms regulatory mechanisms, producing disintegration across these systems.

## Mechanisms, risk, and vulnerability

Several empirical studies offer new insights into how vulnerability to stress-related psychopathology is shaped by developmental, dispositional, and social-environmental factors. First, Inoue et al. [*Mapping research trends in obsessive-compulsive disorder before and after the COVID-19 pandemic: a bibliometric analysis focusing on its molecular mechanisms*] conducted a bibliometric analysis mapping research trends in obsessive-compulsive disorder (OCD). Their main finding is that OCD research is increasingly organized around neurocircuitry, genetics, oxidative stress and inflammation, as well as metabolic interventions, rather than purely descriptive phenomenology, especially after the COVID-19 pandemic. This aligns with the Research Topic's broader emphasis on psychiatric disorders as heterogeneous conditions that require multimodal investigation and treatment.

In addition, Li et al. [*Childhood trauma is associated with perceived stress and hair cortisol levels characterized by the BDNF Val66Met genotype and sex*] showed that the association between childhood trauma and physiological stress responses may be genetically determined and sex-specific. Using a sample of non-clinical young adults (*N* = 190), they found that the relationship between childhood trauma and hair cortisol concentration varied by brain-derived neurotrophic factor (BDNF) Val66Met genotype and sex, with the association more prominent among female Val/Val carriers. This suggests that childhood trauma-related changes in hypothalamic-pituitary-adrenal (HPA) reactivity may be moderated by gene-by-sex processes.

Relatedly, Ecker et al.'s study [*Temperament and personality: preliminary evidence of possible relationships with multifactorial stress reactivity in healthy adolescents*] suggested that heightened stress sensitivity is already reflected in personality and temperament in non-clinical adolescents (*N* = 73). Under experimentally induced social stress, lower extraversion was associated with greater salivary cortisol reactivity, while higher harm avoidance was associated with both stronger cortisol responses and higher subjective stress. These findings suggest that dispositional traits may shape physiological and subjective responses to stressors long before the onset of a psychiatric disorder.

More importantly, stress-related psychopathology may also show meaningful day-to-day temporal variation. Biggs et al. [*Implications for optimizing treatment timing: day of week variation in PTSD symptom clusters*] used ecological momentary assessment to assess PTSD symptoms four times daily for 15 days among U.S. service members with probable PTSD (*n* = 80) and without probable PTSD (*n* = 79). They found that symptom clusters in the probable PTSD group varied across the week and were generally higher on weekdays than weekends, suggesting that symptoms may fluctuate in response to recurring work-related pressures.

Finally, focusing on EEG-derived brain dynamics together with peripheral lactate and lactate dehydrogenase (LDH) in remitted patients with bipolar I disorder (*N* = 20), Kesebir and Demirer [*Serum lactate and LDH are related with theta and gamma activities in bipolar disorder: a band-specific metabolic coupling*] suggested that bipolar I disorder may involve a bioenergetic signature, with serum lactate and LDH linked to theta and gamma EEG activity. This EEG-metabolite association may reflect integrated neuro-energetic and neuroprotective processes that may be relevant to stress sensitivity and longer-term neurodegenerative risk in bipolar I disorder.

## An emphasis on social- and cultural-specific stressors and psychopathology

Social and cultural specificity in the clinical presentation of stressors and psychopathology are highlighted in several empirical studies included in this Research Topic. Region-specific stressors play an important role in mental health difficulties and reduced wellbeing in specific populations. Mohammad et al. [*A study on long-term trauma-related mental health outcomes among Kurdish survivors of chemical attacks*] examined long-term mental health outcomes among survivors of the 1988 chemical attacks in Kurdistan. Among 534 participants, 78.8% met the PTSD threshold, and nearly half (46.3%) showed comorbid somatic and mood symptoms. Greater trauma exposure, female sex, and lower educational attainment were associated with more severe symptoms. These findings suggest that trauma burden and social vulnerability interact such that socially-disadvantaged individuals had a more severe symptom presentation.

Johnson et al. [*Silent scars: understanding interpersonal sensitivity, paranoid ideation, and hostility from adverse childhood experiences in Jamaica*] examined the association between adverse childhood experiences and interpersonal difficulties, including interpersonal sensitivity, paranoid ideation, and hostility, among Jamaican adults (*N* = 1,633). Most participants (70.5%) reported high ACE exposure, and greater ACE burden was associated with worse scores on all three outcomes, with bullying, physical neglect, and home and community violence particularly salient. These findings indicated that early adversity is highly prevalent in Jamaican adults and contributes to later interpersonal difficulties, including paranoia and hostility.

The narrative review of somatic symptom and related disorders in Arab populations by Eid et al. [*Somatic symptom and related disorders in the Arab world: a narrative review of clinical features and care implications*] identified correlates of these disorders in a region where mental health stigma and distinct explanatory models of illness are common. The 35 studies included in the review suggested that, across Arab populations, female sex, lower educational attainment, chronic medical comorbidity, trauma exposure, stigma, and culturally-specific explanatory models influenced symptom expression, help-seeking, ars across multiple domains during psychotherapy rather than restricting evaluation to symptom severity alone. And diagnostic recognition, highlighting the importance of culturally-sensitive understanding of stress-related psychopathology and help-seeking behavior.

Two empirical articles examined country-specific stressors in China. First, Lu et al. [*The longitudinal influence of cyberbullying victimization on depression, self-esteem and academic burnout among Chinese adolescents: mindfulness as a mediator*] found that, among Chinese adolescents (*N* = 1,421), cyberbullying victimization at baseline predicted higher depression and academic burnout, and lower self-esteem at 6-month follow-up. These longitudinal associations were partly mediated by reduced mindfulness. Second, Xue et al. [*Perceived discrimination and subjective wellbeing of left-behind children: social support and psychological resilience as mediators*] found that, among left-behind children in China (*N* = 719), perceived discrimination was cross-sectionally associated with lower subjective wellbeing, both directly and indirectly via lower social support and reduced psychological resilience. A chain mediation pathway from perceived discrimination to diminished support, to reduced resilience, and then to lower subjective wellbeing was evident.

Finally, the data report by Ramos-Galarza et al. [*Stress, anxiety, and depression on traffic police officers: a data report*] found that traffic police officers in Ecuador experience notable levels of stress, anxiety, and depression, reflecting the psychological burden of repeatedly facing dangerous and conflictual work conditions. More broadly, traffic police stress reflects context-specific occupational and social demands, and the most relevant stressors may differ by setting, sociodemographic profile, and broader environmental conditions.

## Intervention implications

Three articles in this Research Topic point toward multilevel, context-sensitive care rather than one-size-fits-all treatment for stress-related psychopathology. Ahari and Fonzo [*A biopsychosocial model of MDMA-assisted therapy in application: dyadic one session treatment for specific phobia*] proposed a biopsychosocial model of 3,4-methylenedioxymethamphetamine (MDMA)-assisted therapy for specific phobia, conceptualized as a dyadic one-session treatment that combines pharmacologic facilitation with psychotherapy. MDMA may reduce fear and defensiveness and enhance trust and emotional openness, thereby making exposure-based work in clinics more effective. More broadly, MDMA-assisted therapy may reshape the social and emotional climate of psychotherapy, making it especially useful for trauma- and fear-related disorders when high arousal and avoidance make exposure difficult.

The validation study of the Systemic Therapy Inventory of Change (STIC) by Brendel et al. [*Translation and validation of the German version of the systemic inventory of change*] underscored the importance of measuring therapeutic indicatos childhood trauma often shapes family dynamics, attachment, and relationship functioning, therapy should address those aspects alongside management of mental health symptoms. Their findings support a broader assessment lens for managing childhood trauma and related psychopathology in therapeutic settings.

Stress-reduction interventions may also be highly relevant in medical settings. Wang et al. [*Effectiveness of psychological intervention based on implicit theory combined with virtual reality in patients with unruptured intracranial aneurysm receiving their first cerebral angiography after flow-diverter stent implantation*] examined the efficacy of a psychological intervention combining implicit theory with virtual reality for patients with unruptured intracranial aneurysm undergoing first cerebral angiography after flow-diverter stent implantation. Their intervention yielded significant reduction in anxiety and improvement in treatment confidence, implicit cognition, physiological regulation, procedural cooperation, and follow-up adherence. The intervention may be a scalable, non-pharmacological adjunct to routine nursing care for reducing procedural stress and improving longer-term adherence.

Overall, the current Research Topic converges on a multilevel approach in which acute and chronic stressors act against the backdrop of pre-existing vulnerabilities to give rise to heterogeneous mental health outcomes in both clinical and non-clinical populations. This broader formulation of stress-induced psychopathology is especially useful for understanding transdiagnostic mental health difficulties. These findings suggest that interventions should be personalized, developmentally-informed, culturally-sensitive, and capable of targeting both stress-induced mechanisms and the vulnerabilities that maintain them.

